# Rethinking Preoperative MRSA/MSSA Screening Through Molecular Triage

**DOI:** 10.3390/diagnostics16152348

**Published:** 2026-07-27

**Authors:** Rob E. Carpenter, Greg Whitlock

**Affiliations:** 1 Soules College of Business, The University of Texas at Tyler, 3900 University Blvd., Tyler, TX 75799, USA; 2 OSPRI Biopath, 7290 Virginia Parkway, Suite 3400, McKinney, TX 75071, USA; gwhitlock@ospribio.com

**Keywords:** MRSA, MSSA, *Staphylococcus aureus*, mupirocin resistance, surgical site infection, diagnostic stewardship, *mecA*, *SCCmec*, preoperative screening, value-based care

## Abstract

**Background:** Preoperative screening for methicillin-resistant *Staphylococcus aureus* (MRSA) and methicillin-susceptible *S. aureus* (MSSA) is intended to identify patients at increased risk of surgical site infection and guide decolonization and perioperative antimicrobial prophylaxis. However, many molecular assays reduce this decision to a binary positive/negative result, potentially obscuring clinically important distinctions in organism identity, methicillin resistance attribution, and mupirocin resistance risk. **Methods:** This structured narrative review organized direct perioperative evidence and indirect mechanistic, implementation, and economic evidence around one question: how MRSA/MSSA screening can move from organism detection to actionable molecular triage. **Results:** Useful preoperative reporting depends on assigning resistance markers to the correct organism. The proposed multi-target NAAT framework organizes concordant and discordant molecular patterns into provisional reportable categories, including MSSA, MRSA, methicillin-resistant non-aureus Staphylococcus/CoNS, mixed populations, *SCCmec* dropout patterns, mupirocin resistance marker states, and invalid or indeterminate results. No externally validated composite score, universal molecular cutoff, or prospectively validated target-to-action decision rule currently links all of these categories to specific perioperative actions. **Conclusions:** Preoperative MRSA/MSSA screening may benefit from moving beyond binary reporting, but the framework presented here is a development-stage rule set rather than a validated clinical decision instrument. Assay-specific analytical thresholds, locked target combination rules, and prospective clinical and implementation validation are required before the framework can be used to assign patients reproducibly to management pathways. Until such validation is completed, the proposed categories should be interpreted as a testable reporting and validation architecture rather than as universal prophylaxis or decolonization instructions.

## 1. Introduction

Preoperative screening for methicillin-resistant *Staphylococcus aureus* (MRSA) and methicillin-susceptible *S. aureus* (MSSA) colonization is often framed as a discrete laboratory cost. Under this view, the test appears financially weak when it is not directly reimbursed. Yet this framing misses the functional role of screening in high-risk surgical pathways. In procedures involving prosthetic material, such as total hip and knee arthroplasty, the relevant problem is not simply whether the preoperative test is paid for. The relevant problem is whether microbial information obtained before incision can reduce the probability that colonization becomes postoperative infection [1]. In this setting, screening has value only insofar as it helps organize a chain of action: targeted decolonization, antimicrobial prophylaxis, infection prevention planning, and episode-level risk reduction [2,3]. The economic unit is therefore not the test. It is the surgical episode.

The clinical premise is well established. *S. aureus* colonization, particularly nasal carriage, increases the risk of subsequent infection, including surgical site infection and, ultimately, serious bloodstream events [4]. Decolonization regimens combining intranasal mupirocin with chlorhexidine bathing have supportive evidence in selected surgical and other high-risk populations [5], but the evidentiary pathway is not uniform across settings. Perioperative guidance supports preoperative anti-staphylococcal decolonization for selected high-risk procedures, whereas ICU and post-discharge MRSA decolonization studies should be interpreted as indirect evidence when applied to preoperative SSI prevention [6,7,8]. Current prevention guidance supports preoperative anti-staphylococcal decolonization for orthopedic and cardiothoracic procedures and other high-risk procedures involving prosthetic material [6,7,9,10,11]. A 2024 ESCMID/EUCIC guideline, developed through systematic review and formal GRADE assessment, similarly recommends *S. aureus* screening and decolonization before selected high-risk operations while emphasizing antimicrobial stewardship involvement and the limited evidence available for methicillin-resistant CoNS [11]. The literature published from 2021 through 2026 was prioritized when contemporary evidence was available, particularly for clinical implementation, molecular diagnostics, diagnostic stewardship, resistance epidemiology, and perioperative prevention. Earlier publications were retained when they represented foundational studies, established biological mechanisms, or remained the principal evidence supporting a specific analytical limitation.

The unresolved issue is therefore not whether colonization matters, or whether decolonization can reduce risk. The issue is how preoperative MRSA/MSSA screening should be valued, designed, and reported when its benefit is expressed indirectly. Screening acts upstream, but its consequences appear downstream: in risk stratification, prophylaxis selection, decolonization success, avoided infection, reduced antimicrobial exposure, shorter length of stay, fewer readmissions, and improved episode-level performance. This creates a mismatch between where the cost is visible and where the value is realized.

A similar logic applies to antimicrobial prophylaxis. Current guidance distinguishes routine procedure-based β-lactam prophylaxis from MRSA-directed prophylaxis. Vancomycin is not recommended for routine prophylactic use, but it may be appropriate for patients known to be MRSA-colonized, including those identified through preoperative screening, particularly when prosthetic material is involved [6,7]. This creates a practical diagnostic problem. If prophylaxis and decolonization depend on microbial status, then the laboratory report must do more than announce a binary MRSA-positive or MRSA-negative result [12].

Molecular reporting can obscure clinically important distinctions by compressing different organism resistance states into a single MRSA category. A report may detect *S. aureus*; *mecA/mecC*, an *SCCmec*-associated signal; or a mixed staphylococcal background, but these signals do not always have the same clinical meaning. Methicillin resistance attributable to *S. aureus* is not equivalent to *mecA* carried by coagulase-negative staphylococci. An *SCCmec*-*orfX* signal without an intact internal resistance determinant is not the same as confirmed MRSA colonization. Mupirocin resistance changes the decolonization pathway, but it does not by itself justify MRSA-directed β-lactam avoidance. The central problem is therefore not analytical detection alone. It is interpretive translation.

This review proposes that preoperative MRSA/MSSA testing should be designed and reported as molecular triage. In this model, the result is not merely positive or negative. It is an organism–resistance–decolonization interpretation that answers three questions before incision: Is *S. aureus* present? Is methicillin resistance attributable to *S. aureus* rather than coagulase-negative staphylococci (CoNS) or another staphylococcal background? Is mupirocin likely to remain an appropriate decolonization agent, or does the molecular pattern indicate elevated risk of decolonization failure? Framed this way, the report becomes part of the intervention. It links microbial state to perioperative action.

### Purpose and Scope of This Narrative Review

This narrative review undertakes a conceptual and translational task rather than a clinical outcome claim. Its purpose is not to establish that any specific MRSA/MSSA-MupR assay has been clinically validated to reduce surgical site infection, nor to offer universal prophylaxis or decolonization instructions. Instead, the review defines the interpretive logic that a next-generation preoperative molecular screening strategy would need to satisfy before such clinical claims could be tested. The central undertaking is to show how organism identity, methicillin resistance attribution, *SCCmec* or flanking region signals, CoNS background, mupirocin resistance markers, body site context, and internal control status can be organized into clinically meaningful categories.

The review therefore treats the laboratory report as a candidate decision architecture. In this architecture, molecular targets are not interpreted as isolated positives or negatives. They are organized into provisional organism–resistance classifications, classification status categories, candidate pathway implications, reflex testing needs, and validation requirements. This distinction is critical because the same molecular signal may have different clinical meaning depending on organismal context. *mecA/mecC* in a CoNS background does not establish MRSA colonization. *SCCmec*-*orfX* positivity without *mecA/mecC* does not confirm methicillin-resistant *S. aureus*. *mupA/mupB* modifies decolonization logic but does not, by itself, justify MRSA-directed prophylaxis.

Accordingly, the contribution of this review is a framework for interpretive molecular triage. The framework is intended to clarify what should be reported, how discordant or mixed target patterns should be classified, what clinical actions should and should not follow from those patterns, and what analytical, clinical, implementation, and economic validation would be required before the framework could be adopted as a validated perioperative intervention. Importantly, the framework proposed in this review is not a validated scoring system. Based on currently available perioperative guidance and assay-specific molecular labeling, no universal numerical score or cross-platform cycle threshold cutoff has been established for translating the combined *S. aureus*, non-aureus Staphylococcus/CoNS, *mecA/mecC*, *SCCmec* flanking, mixed population, and mupirocin resistance marker patterns into perioperative actions. Existing clinical guidance generally links action to established categories such as documented *S. aureus* or MRSA colonization, while commercial molecular platforms use device-specific analytical calling rules. The framework presented below should therefore be understood as a provisional categorical classification and validation schema. It is intended to standardize testable report categories, not to authorize autonomous treatment decisions before assay-specific analytical validation, prospective clinical validation, and institutional protocol approval.

## 2. Method

This narrative review [13] is organized around a single translational problem: how preoperative MRSA/MSSA screening can be transformed from a laboratory classification into a perioperative decision signal. A narrative review design is appropriate because the question addressed here is not limited to a single intervention effect estimate. Rather, it requires synthesis across microbiology, perioperative prophylaxis, decolonization, molecular assay design, diagnostic stewardship, antimicrobial stewardship, infection prevention, implementation science, and value-based surgical care. In this setting, the relevant problem is not only whether screening detects colonization, but whether the result can be interpreted and acted upon within the clinical pathway in which SSI risk is produced.

This review therefore uses a structured narrative approach rather than a pooled meta-analysis. Narrative reviews are useful when the literature spans heterogeneous study designs, populations, mechanisms, and implementation contexts, particularly when the purpose is conceptual integration rather than quantitative effect estimation [14,15,16]. To preserve rigor, the evidence is organized by clinical function and epistemic proximity to the preoperative surgical screening question. Direct perioperative and surgical site infection evidence is used to support claims about SSI prevention, prophylaxis, and decolonization pathways. ICU, post-discharge, pediatric, neonatal, and modeling studies are used more cautiously as indirect mechanistic or implementation evidence. These sources identify plausible variables that may matter for preoperative reporting, but they are not treated as proof that an MRSA/MSSA-MupR reporting strategy reduces SSI.

### Classification of Evidence Directness and Strength

Because this was a structured narrative review rather than a formal systematic review, the Grading of Recommendations Assessment, Development and Evaluation framework was not applied and pooled certainty estimates were not generated. To make evidentiary heterogeneity explicit, each principal component of the proposed framework was classified along two dimensions: directness to adult preoperative surgical screening and strength of support. Direct evidence included perioperative studies or guidelines involving surgical populations and clinically relevant perioperative outcomes. Partially direct evidence included surgical or perioperative studies addressing related populations, interventions, or outcomes. Indirect evidence included ICU, post-discharge, pediatric, neonatal, computational modeling, and cross-diagnostic workflow studies. Conceptual evidence denoted author-derived target combinations, reporting rules, or pathway mappings that have not undergone prospective clinical validation. Evidence strength was described as higher, moderate, limited, or very limited according to study design, consistency, population relevance, and proximity to the proposed use. These classifications are narrative descriptors and should not be interpreted as formal GRADE ratings.

The review brings together six domains of evidence that are often considered separately but are clinically interdependent. The first domain concerns the biological link between *S. aureus* colonization and SSI risk, with attention to how preoperative decolonization modifies that risk. The second domain concerns perioperative antimicrobial prophylaxis, particularly the way MRSA colonization status informs vancomycin use, β-lactam preservation, and procedure-specific prophylaxis decisions. The third domain situates screening within value-based surgical care, where the relevant unit of analysis is not the reimbursed test but the surgical episode, including hospital-acquired condition policy, readmission risk, length of stay, bundled payment logic, and avoidable downstream cost.

The organizing premise is diagnostic stewardship. Diagnostic stewardship emphasizes not only the ordering of the right test for the right patient, but also the reporting and interpretation of results in a way that prompts the right clinical action [17]. This is directly relevant to preoperative MRSA/MSSA screening because the same molecular signal can have different clinical meanings depending on organismal context. *mecA/mecC* detected in *S. aureus* supports an MRSA colonization pathway; *mecA* detected in coagulase-negative staphylococci does not establish MRSA colonization; mupirocin resistance markers may redirect decolonization but do not, by themselves, justify MRSA-directed prophylaxis. The review therefore treats the laboratory report as part of the intervention pathway rather than as a passive output after testing.

The review also develops a practical assay interpretation architecture derived from a proposed two-well MRSA/MSSA-MupR panel design. The full hypothesis-generating target pattern logic is provided in Appendix A, with the proposed molecular classification and reporting architecture presented in condensed form below. In this framework, organism identity, *SCCmec*-*orfX* junction detection, *mecA/mecC*, *spa* or comparable *S. aureus* redundancy, *mupA/mupB*, selected *ileS* markers when validated, and assay controls are not treated as isolated molecular outputs. They are mapped into clinically meaningful triage categories that distinguish MSSA colonization, MRSA colonization, methicillin-resistant CoNS, empty cassette or dropout patterns, mupirocin resistance risk, and invalid or indeterminate results. This architecture is intended to make the report clinically interpretable, not merely analytically positive or negative.

The objective is not to claim that an unvalidated molecular panel independently reduces SSI risk. Rather, the objective is to define the scientific, clinical, and reporting logic that a next-generation preoperative MRSA/MSSA-MupR assay must satisfy before such a claim could be tested. The central claim is that clinical utility emerges only when molecular detection is coupled to interpretation: when the report assigns resistance to the correct organism, separates prophylaxis decisions from decolonization decisions, identifies resistance-mediated decolonization risk, and supports implementation within a real surgical pathway. In this sense, the assay is treated not simply as a test, but as a candidate decision architecture for diagnostic stewardship, antimicrobial stewardship, infection prevention, and value-based surgical care.

Because several cited studies were conducted outside the preoperative surgical-screening context, this review explicitly distinguishes direct perioperative evidence from indirect mechanistic or implementation evidence. Studies of ICU decolonization, postdischarge MRSA clearance, pediatric or neonatal surveillance, and computational modeling are used to identify plausible biological and operational variables relevant to preoperative reporting, not to claim that MRSA/MSSA-MupR reporting has already been shown to reduce SSI. The proposed framework therefore remains a translational and validation agenda rather than a proven clinical-outcome intervention. Studies of molecular triage in bloodstream and respiratory infections were also examined as cross-setting implementation precedents, with emphasis on how assays were validated, linked to stewardship or clinical pathways, and evaluated for treatment and workflow effects.

For implementation purposes, this review separates molecular classification from clinical pathway assignment. In the first stage, the laboratory assigns a reportable molecular category through locked, prespecified target combination rules. In the second stage, the clinical team maps that category to an institution-approved pathway that also incorporates procedure type, prosthetic material exposure, allergy status, time to surgery, age or chlorhexidine eligibility, contraindications, and local epidemiology. Clinicians should not be expected to combine raw cycle threshold or fluorescence values at the bedside. Target-specific positivity cutoffs, internal control acceptance limits, equivocal or repeat zones, and reflex testing criteria must be established for the specific assay, instrument, specimen type, and intended use during validation. No universal Ct threshold or weighted clinical score is proposed in this review.

## 3. Results

### 3.1. The Value-Based Imperative: Selling Against “Doing Nothing”

The practical barrier to preoperative MRSA/MSSA screening is rarely a superior competing assay. It is the inertia of non-adoption. From the hospital’s perspective, routine screening creates a visible and immediate cost, while the event it seeks to prevent is probabilistic, delayed, and distributed across the surgical episode. This produces a prevention value mismatch: the cost of screening is certain, but the value of screening becomes visible only if downstream infection risk is reduced.

Value-based and episode-based payment models change the meaning of that mismatch. Under hospital-acquired condition policy, the Hospital-Acquired Condition Reduction Program (HACRP) reduces Medicare payments for hospitals with poor performance on specified hospital-acquired condition measures, including healthcare-associated infection measures. The Hospital Readmissions Reduction Program (HRRP) adjusts payment for excess 30-day readmissions and includes elective primary total hip and/or total knee arthroplasty among its covered procedure-specific measures. Although the original Comprehensive Care for Joint Replacement model ended in 2024, it remains an important policy precedent because it treated lower-extremity joint replacement as an episode of care rather than as a set of isolated departmental transactions.

This policy context reframes the economic question. The issue is not simply whether the screening test is reimbursed as a discrete service. The issue is whether the absence of preoperative microbial information permits preventable SSI-related costs to propagate through the episode [18]. Surgical site infection can increase length of stay, readmission risk, antimicrobial exposure, revision burden, post-acute utilization, and total cost. It was estimated that SSI added approximately 11.2 hospital days and $20,785 per case, while MRSA-associated SSI added approximately 23.0 days and $42,300 per case [19]. In a separate national cost analysis [20], it was found that SSI extended length of stay by 9.7 days, increased cost by $20,842 per admission, and generated substantial readmission-related utilization and excess cost.

These estimates should not be converted into a deterministic return-on-investment claim for any single test. Screening does not mechanically prevent SSI. Rather, these figures define the risk environment in which screening may become valuable. A preoperative microbial result has financial meaning only when it improves the reliability of a prevention system: identifying colonized patients, directing decolonization, informing prophylaxis, reducing inappropriate vancomycin use, and supporting infection prevention coordination before incision [21].

This distinction is central to the argument of this review. Screening is not a stand-alone intervention. It is a control point within a broader perioperative prevention pathway that includes hand hygiene, chlorhexidine bathing, nasal decolonization, antimicrobial prophylaxis selection, environmental cleaning, patient adherence, isolation practices when indicated, and post-discharge surveillance [21]. If these downstream components fail, screening alone cannot rescue the episode. However, without accurate preoperative microbial information, the bundle becomes less targeted. Prophylaxis and decolonization decisions are more likely to follow institutional habit than patient-specific risk. In value-based surgical care, doing nothing is therefore not neutral. It is a decision to manage perioperative infection risk with less information than the episode may require [22].

### 3.2. Surgical Site Infection Outcomes Under Decolonization Protocols

Surgical decolonization studies support a central premise of this review: preoperative *S. aureus* screening is not valuable because it detects colonization in isolation. It is valuable when detection changes what the perioperative system does next. Screening does not prevent infection by itself. It becomes clinically meaningful only when colonization status is translated into a coordinated sequence of decolonization, prophylaxis selection, adherence monitoring, and infection prevention action [23].

The Ontario Health technology assessment supports this bundle-based interpretation [23]. Nasal mupirocin combined with chlorhexidine body wash was found to reduce *S. aureus*-related surgical site infection risk among *S. aureus* carriers undergoing selected high-risk procedures, including cardiothoracic, vascular, orthopedic, gastrointestinal, and general surgery [23,24]. The strongest targeted decolonization evidence showed reduced *S. aureus*-related SSI risk when carriers received mupirocin plus chlorhexidine body wash compared with placebo [5,24]. The same assessment also found that both universal and targeted decolonization may reduce *S. aureus*-related SSI compared with no decolonization, although the economic result depends on screening cost, baseline *S. aureus* prevalence, SSI treatment cost, and mupirocin efficacy [24]. Thus, the relevant intervention is not the test alone, but the test-linked prevention pathway [8,23].

Recent orthopedic and spine surgery data illustrate the same principle while also showing its boundary conditions. In adults undergoing arthroplasty or spine surgery under a protocol of preoperative MRSA/MSSA testing, daily chlorhexidine cleansing, mupirocin for MRSA/MSSA carriers, and restricted vancomycin use, MRSA carriage remained associated with SSI, whereas MSSA carriage was not associated with SSI [25]. Nonadherence to chlorhexidine or mupirocin was documented in two of three *S. aureus* carriers who developed *S. aureus* SSI. The implication is not that screening lacks value. Rather, it is that screening value is conditional: the result must activate, and the patient must complete, the correct decolonization and prophylaxis pathway.

Orthopedic trauma and fracture fixation data make this implementation constraint even more visible. For example, a recent evaluation of adults undergoing operative fixation for acute pelvic or extremity fractures at a Level 1 trauma center assessed a quality-improvement protocol requiring *S. aureus* nasal screening and targeted carrier decolonization with intranasal mupirocin and chlorhexidine bathing [26]. Among screened first procedures, 20.29% were performed on MSSA carriers and 4.90% on MRSA carriers. After adjustment, MRSA carriage was significantly associated with any SSI, *S. aureus* SSI, and MRSA SSI, whereas MSSA carriage was not significantly associated with these outcomes. The adjusted odds ratios were 4.58 for any SSI, 10.11 for *S. aureus* SSI, and 27.25 for MRSA SSI [26]. Importantly, the message from the fracture fixation cohort is not only microbiological. It is temporal. Although the decolonization protocol required five days, 75.57% of procedures performed on *S. aureus* carriers occurred within four or fewer days after injury, making completion of the full mupirocin protocol difficult in urgent surgical pathways [26]. Documented mupirocin use occurred in only 40.28% of MSSA carrier procedures and 64.71% of MRSA carrier procedures [26]. These findings sharpen the implementation thesis of this review: detection accuracy is necessary, but insufficient. A screening strategy becomes clinically actionable only when the report, decolonization protocol, prophylaxis pathway, and surgical timeline can be made to operate together before incision.

These studies move the argument from detection to control. The problem is not simply whether *S. aureus* is present. The problem is whether the health system can use that information fast enough, specifically enough, and reliably enough to alter infection risk. A next-generation preoperative MRSA/MSSA-MupR report should therefore be judged not only by analytical performance, but by whether it improves the execution of the prevention bundle in the real perioperative pathway.

### 3.3. Decolonization Effectiveness and Implementation Realism

Mupirocin resistance interpretation increases the clinical value of a preoperative MRSA/MSSA report, but it should not be mistaken for a complete model of decolonization success. Decolonization is not a binary consequence of detecting colonization or resistance. It is a pathway-dependent intervention whose success depends on the interaction between microbial biology, body site distribution, patient behavior, and protocol execution [27,28]. The relevant questions are therefore broader than whether MRSA or MSSA is present. Which body sites are colonized? Does the regimen reach those sites? Can the patient complete the protocol before surgery? Are household or environmental reservoirs relevant? Is the organism susceptible to the selected agent? A molecular result improves this pathway only when it helps organize these decisions rather than replace them.

This distinction is especially important in urgent fracture fixation pathways. In these settings, the interval between injury and surgery may be shorter than the conventional five-day mupirocin protocol, even when MRSA or MSSA carriage is correctly identified [28]. Thus, the problem is not only whether screening detects the organism. The problem is whether the detection event occurs early enough, and in a form actionable enough, to change the perioperative pathway. This logic is consistent with outcome evidence showing that mupirocin-plus-chlorhexidine decolonization reduces *S. aureus*-related SSI most clearly when applied to confirmed *S. aureus* carriers, while real-world performance still depends on adherence, patient selection, and local implementation fidelity [24,25].

Recent implementation evidence reinforces this broader interpretation. In a retrospective outpatient MRSA decolonization study with a 12- to 15-month follow-up, 72 of 92 successfully followed carriers (78.3%) achieved long-term MRSA clearance [29]. Yet, decolonization failure was associated with carriage complexity. Throat carriage with other colonized sites remained significant in multivariate analysis, and a higher number of colonized sites increased the likelihood of failure [29]. This finding is directly relevant to preoperative reporting. Absence of a detected high-level mupirocin resistance marker does not guarantee successful decolonization when colonization is multisite, extranasal, or operationally difficult to eradicate.

Pediatric evidence illustrates the same principle from another implementation context [30]. A recent summary of pediatric *S. aureus* hygiene and decolonization evidence demonstrated that while protocols often combine intranasal mupirocin with chlorhexidine washes or bleach baths, recurrence outcomes remain inconsistent and compliance serves as a major limiting factor [31]. This evidence is useful because it separates drug selection from pathway success. Decolonization depends not only on the agent chosen, but also on household burden, patient and family adherence, health literacy, hygiene practices, and whether the intervention is limited to the index patient or extended to household contacts [31,32].

The perioperative relevance of implementation design is further illustrated in a recent study of 190 pediatric cardiac surgery patients that compared routine preoperative care with a preoperative decolonization protocol using chlorhexidine gluconate (CHG) washing and intranasal mupirocin [33]. In this cohort, the intervention group demonstrated lower preoperative nasal and axillary MRSA and coagulase-negative staphylococcal colonization, fewer postoperative wound infections, and shorter ICU stays [33]. While these findings should not be overgeneralized to adult orthopedic pathways, their importance is conceptual and operational: they demonstrate that skin and nasal decolonization can reduce perioperative staphylococcal burden when implemented as a defined protocol rather than treated as the passive consequence of a test result.

These findings sharpen the central claim. A next-generation MRSA/MSSA-MupR report should not imply that molecular detection alone determines outcome. It should identify the organism, clarify resistance risk, and support the next action in a pathway whose success also depends on timing, anatomy, adherence, household ecology, and local protocol design. Decolonization fails not only when the organism is resistant, but also when the pathway cannot reach, sustain, or complete the intervention.

### 3.4. Age-Stratified and Neonatal Implementation Considerations

Neonatal and pediatric decolonization evidence clarifies a central principle of this review: MRSA/MSSA-MupR reporting should not be interpreted as a universal command to decolonize. It should function as population-specific clinical triage. The same molecular pattern can have different clinical meaning depending on age, body site distribution, safety constraints, protocol feasibility, and the purpose of surveillance. In adult elective surgery, a positive result may be used to prevent SSI before incision. In neonatal intensive care units and young pediatric populations, the same result may instead serve outbreak control, transmission reduction, or longitudinal surveillance. Thus, organism detection is only the first step. The report must also help determine whether the detected state is actionable in the patient’s clinical context.

This distinction is especially important in neonates. Colonized infants may be premature, may fall below gestational age or chronological age thresholds for CHG exposure, may be discharged before a decolonization protocol can be completed, or may be managed under unit-level infection control assumptions rather than adult preoperative SSI prevention assumptions. Neonatal and pediatric evidence should therefore be understood not as direct proof for adult orthopedic SSI prevention, but as an age-stratified extension of the same reporting logic: molecular detection becomes useful only when it is mapped onto a feasible and safe intervention pathway.

The Texas Children’s Hospital neonatal intensive care unit (NICU) experience illustrates this implementation problem. One recent study evaluated a 42-bed level III NICU before and after the implementation of weekly MRSA surveillance and targeted decolonization [34]. Screening occurred at birth or admission and weekly thereafter until discharge, utilizing nares, axilla, and groin specimens. Under this protocol, MRSA-colonized neonates received intranasal mupirocin, while CHG wipes were restricted to infants meeting specific age-based safety criteria (at least 4 weeks postnatal age or 36 weeks gestational age). Following implementation, the rate of MRSA infection decreased, with no MRSA infections developing in colonized neonates who received both mupirocin and CHG [34]. Because baseline MRSA infection prevalence was low, these findings should not be interpreted as a universal neonatal decolonization mandate. Rather, their importance is operational: they demonstrate that the clinical value of a positive MRSA result hinges on the successful coordination of surveillance cadence, multisite sampling, age-based eligibility rules, electronic order sets, and protocolized decolonization within a functioning NICU workflow [34].

A complementary lesson regarding system-level interventions highlights this operational nuance. When renewed MRSA surveillance and decolonization efforts—incorporating protocol revisions, electronic medical record order set updates, staff re-education, and weekly leadership reviews—were implemented in a level IV NICU, they were associated with a sustained decrease in mean MRSA bloodstream infection rates, from 0.10 to 0.03 per 1000 patient-days [35]. Crucially, however, a parallel MSSA surveillance and decolonization program did not produce a statistically significant decrease in MSSA bloodstream infection rates [35]. This organism-specific difference is important: although MRSA and MSSA appear analytically parallel in a molecular report, they cannot be assumed to function as identical implementation targets. Therefore, a mature reporting architecture must allow for MRSA and MSSA to have different action thresholds, different prevention logic, and different evidentiary strength supporting their intervention pathways [35].

Evidence from pediatric populations further cautions against automatic decolonization following a positive MRSA result. A retrospective follow-up evaluation of MRSA carriers younger than 6 years revealed that decolonization treatment provided no significant added benefit over no treatment for children under 2 years or those aged 2–5 years [36]. These results support a wait-and-see approach for children under 2 years—and potentially for those up to 6 years of age—while acknowledging the need for further investigation [36]. This reinforces the critical distinction between detection and intervention: a positive MRSA result might necessitate decolonization in one context, yet only warrant surveillance, delayed intervention, or outbreak control measures in others. Ultimately, age, local prevalence, spontaneous clearance, household transmission, and protocol feasibility dictate the practical implications of a positive result [36].

Neonatal and pediatric studies do not weaken the adult preoperative screening argument. They define its boundary conditions. A next-generation MRSA/MSSA-MupR report should not merely label a specimen as MRSA-positive, MSSA-positive, or mupirocin-resistant. It should help determine whether the result is actionable for the patient population and clinical pathway in which it appears. For neonatal and pediatric use, that means separating organism resistance interpretation from decolonization eligibility, CHG safety criteria, gestational age, chronological age, discharge timing, skin integrity, household transmission risk, electronic order set activation, multisite or extranasal carriage, protocol completion, and adherence review. When high-level MupR markers are detected, the result may justify evaluation of an institution-approved non-mupirocin pathway; however, the supporting pediatric and neonatal evidence remains indirect for adult preoperative use [37]. When such markers are absent, the report should avoid implying guaranteed decolonization success. Instead, it should frame mupirocin as microbiologically reasonable only when the patient is eligible for, and able to complete, the decolonization bundle [38].

## 4. Discussion

### 4.1. Clinical Utility: From Colonization to Perioperative Action

Preoperative MRSA/MSSA screening becomes clinically useful when it transforms colonization biology into perioperative action. The value of the report is therefore not exhausted by the detection of *S. aureus*. A clinically mature report should answer three linked questions before incision: Is the patient colonized with MSSA or MRSA? If methicillin resistance is detected, is that resistance attributable to *S. aureus* rather than to coagulase-negative staphylococci or another staphylococcal background organism? If decolonization is indicated, does the molecular pattern support mupirocin use, or does it suggest that an alternative pathway may be needed?

This distinction matters because MSSA and MRSA occupy different positions in the perioperative decision space. MSSA colonization may justify *S. aureus*-directed decolonization, particularly in procedures where *S. aureus* SSI prevention is clinically consequential. However, MSSA colonization does not generally require MRSA-directed prophylaxis when standard perioperative β-lactam prophylaxis remains appropriate. MRSA colonization changes the prophylaxis problem because published guidance discourages routine vancomycin use while supporting MRSA-active prophylaxis, commonly vancomycin, for patients known to be MRSA-colonized or in institutionally defined high-risk settings [6]. The report must therefore distinguish MSSA from MRSA and distinguish both from CoNS or other staphylococcal background organisms.

Culture-based MRSA screening remains available and may be less technology-intensive, but its clinical limitation is temporal. Conventional culture introduces delay and can be influenced by incubation time, enrichment strategy, resistance expression dynamics, and medium-specific detection limitations. Polymerase chain reaction (PCR)-based screening reduces turnaround time and enables rapid molecular target detection. In a prospective crossover study of surgical wards, patients were 1.49 times more likely to acquire MRSA during the culture-based screening period than during the rapid PCR period, and positive-result reporting was faster with rapid testing than with culture-based screening [39]. Meta-analytic evidence also supports strong diagnostic performance for PCR-based MRSA testing, although sensitivity and specificity vary by platform, specimen type, comparator method, and culture incubation conditions [40]. The point is not that PCR is intrinsically superior in every setting. The point is that rapid molecular testing creates a window for action only if the result is interpreted correctly.

The central interpretive problem is that *mecA/mecC* detection alone does not equal MRSA. CoNS commonly colonize skin and mucosal surfaces and may carry methicillin-resistance determinants. A nasal specimen containing methicillin-resistant CoNS without *S. aureus* is not the same clinical state as MRSA colonization. If this pattern is overcalled as MRSA, the result may drive unnecessary MRSA-directed prophylaxis, excess vancomycin exposure, avoidable pharmacy burden, and distorted infection prevention surveillance. Conversely, if *S. aureus* is detected but methicillin resistance is missed or not attributed correctly, true MRSA colonization may be undertreated.

A second interpretive problem is the empty cassette or mecA dropout pattern. Some *S. aureus* strains contain *SCCmec* remnants or *SCCmec*-*orfX* junction sequences without an intact internal mecA resistance determinant. Assays that rely too heavily on a single *SCCmec*-*orfX* junction signal can therefore generate false-positive MRSA calls when the resistance gene itself is absent [41,42,43]. This is not a trivial analytical nuance. False-positive MRSA results can lead to unnecessary isolation, decolonization, and glycopeptide use [41]. Assay design should therefore treat *SCCmec*-*orfX*, *mecA/mecC*, and *S. aureus* identity as interdependent signals rather than interchangeable proxies.

A third problem is target variability. *spa* is useful because staphylococcal protein A is strongly associated with *S. aureus* and can add organism-level redundancy. Yet no single target should be treated as infallible. A robust assay architecture should use multiple independent signals to reduce both false-positive and false-negative interpretation [12]. In practical terms, *S. aureus* detection, a redundant *S. aureus* target such as *spa*, *SCCmec*-*orfX*, *mecA/mecC*, and CoNS/*Staphylococcus* spp. context should be interpreted together. When these signals are concordant and satisfy a validated assay-specific rule, they may support a resolved molecular classification. When they are discordant, the report should preserve the uncertainty and assign a provisional or indeterminate classification requiring repeat or reflex testing.

The most clinically useful extension of MRSA/MSSA screening is mupirocin resistance interpretation. Universal and targeted decolonization protocols often rely on intranasal mupirocin, usually combined with chlorhexidine bathing [44]. However, mupirocin resistance can undermine decolonization and may be selected by repeated or widespread exposure [44,45,46,47]. High-level mupirocin resistance is most often mediated by *mupA*, with *mupB* also described, whereas low-level resistance is associated with chromosomal mutations in *ileS*, including mutations affecting the Rossmann fold such as V588F and V631F [46,47,48]. A report that identifies *S. aureus* but does not address mupirocin resistance marker status leaves the pharmacist and surgeon with an untested assumption [49,50]. This is clinically and economically relevant because the value of decolonization strategies is sensitive to mupirocin efficacy, and reduced effectiveness from resistance or poor adherence can materially alter the value of both targeted and universal decolonization strategies [24].

Recent genomic data further sharpen this concern. In a recent genomic analysis of 384 *S. aureus* clinical isolates collected in Tampa, Florida from 2017 through 2023, mupirocin resistance was observed in 106 isolates and was predominantly concentrated among MRSA [51]. Specifically, 103 of the 106 mupirocin-resistant isolates were MRSA, accounting for 34.4% of the collection’s MRSA cohort. Notably, overall mupirocin resistance prevalence remained stable between the pre-COVID-19 and post-COVID-19 periods, even after a hospital protocol shifted from mupirocin to povidone-iodine decolonization [50]. Mechanistically, high-level resistance was strongly associated with mupA, while low-level resistance was driven by *ileS* mutations, including V588F and novel variants. The concurrent discovery of a high-prevalence ST3390 MRSA-MupR group highlights a critical operational takeaway: local molecular epidemiology is a defining factor in determining whether mupirocin remains a rational default therapy [51].

For preoperative triage, the implication is direct. MSSA with a detected mupirocin resistance marker does not necessarily require MRSA-directed prophylaxis, but it may require a non-mupirocin decolonization pathway. MRSA with no detected mupirocin resistance marker may require MRSA-directed prophylaxis and standard mupirocin-based decolonization, assuming the patient is eligible for and able to complete the protocol. MRSA with a mupirocin resistance marker may require both MRSA-directed prophylaxis and an alternative decolonization strategy. The report should therefore not merely state “*mupA* detected.” It should explain that the finding increases concern for mupirocin decolonization failure and should direct the user toward an institution-approved alternative pathway.

Comparative effectiveness evidence further supports this caution. A large, pragmatic cluster-randomized noninferiority trial involving 801,668 adult ICU admissions across 137 hospitals compared universal iodophor–CHG decolonization with continued universal mupirocin–CHG decolonization [8]. The results showed that iodophor–CHG did not meet noninferiority criteria and was consistent with inferiority for *S. aureus* and MRSA clinical cultures, though bloodstream infection outcomes did not differ significantly [8]. While not a preoperative SSI trial, this evidence provides indirect caution against assuming that iodophor-based nasal antisepsis is automatically equivalent to mupirocin-based decolonization for anti-staphylococcal outcomes. Consequently, when MupR is detected, molecular reports should direct clinicians toward a locally validated alternative rather than implying that any non-mupirocin nasal agent is functionally interchangeable [8].

These findings define the clinical utility of preoperative molecular screening. The proposed architecture extends beyond gene detection by testing whether molecular signals can be assigned to organisms, organisms can be classified into provisional molecular states, and validated classifications can subsequently support institution-approved perioperative pathways. A next-generation MRSA/MSSA-MupR report should therefore function as an interpretive interface between molecular biology and surgical decision-making. Its purpose is to reduce avoidable ambiguity before incision: when to decolonize, when to preserve β-lactam prophylaxis, when to add MRSA-active coverage, when to avoid mupirocin, and when to request reflex clarification because the molecular pattern is biologically plausible but clinically indeterminate.

### 4.2. Mechanistic Modeling of Body Site Dynamics and Resistance-Mediated Decolonization Failure

Mechanistic modeling strengthens a central claim of this review: decolonization should not be treated as a binary action that follows automatically from MRSA detection. It is better understood as a site-specific and resistance-sensitive control problem. A report that states “MRSA detected” is therefore incomplete unless it also helps the clinician infer where colonization is located, whether the organism is likely to respond to mupirocin, and whether the detected molecular state increases the probability of decolonization failure.

Computational modeling provides an indirect rationale for considering body site context in decolonization efforts. When a Bayesian coupled hidden Markov model was applied to longitudinal MRSA carriage data from a post-discharge decolonization setting, the nares emerged as a central hub in MRSA colonization dynamics [52]. Furthermore, this analysis estimated nasal mupirocin to be the single most important component of therapy, even though the complete protocol outperformed any isolated component [52]. If these site-specific dynamics hold true for preoperative *S. aureus* carriers, multisite or nares-positive carriage could serve as a clinically relevant modifier of decolonization failure risk; however, this inference still requires direct preoperative validation [52].

Complementary indirect evidence on resistance-mediated clearance failure has also emerged from recent Bayesian modeling of sequenced MRSA isolates [53]. Within a decolonization cohort, this analysis revealed that high-level mupirocin resistance determinants were strongly associated with protocol failure, whereas putative chlorhexidine resistance markers and resistance to unrelated antibiotics showed no such relationship [53]. Should this resistance–clearance dynamic hold true in preoperative screening populations, *mupA/mupB*-mediated high-level resistance would warrant greater interpretive weight than less validated antiseptic tolerance markers. Until direct validation is achieved, however, MupR should be reported strictly as a decolonization risk modifier rather than a definitive predictor of preoperative SSI prevention failure [53].

These modeling studies move the report from detection to inference. *S. aureus* identity, methicillin resistance assignment, mupirocin resistance status, and body site distribution should be interpreted as linked variables rather than independent results. A nares-positive, high-level mupirocin-resistant MRSA pattern may identify a candidate decolonization failure state; however, this inference is derived primarily from indirect modeling evidence and requires direct preoperative validation. Conversely, detection of putative antiseptic tolerance markers should not automatically override a mupirocin-based pathway unless the institution has demonstrated that those markers predict failure under its own decolonization protocol. The proposed interpretive model therefore requires two linked levels: the analytical layer detects organism and resistance targets. The interpretive layer maps target patterns to preoperative action.

### 4.3. Assay Target Architecture and Interpretive Logic

The proposed two-well architecture is presented as a development-stage rule-based classification framework, not as a validated clinical decision algorithm. Its purpose is to define testable target combination rules and the validation required before those rules can support clinical pathway assignment. The first well establishes the staphylococcal background and decolonization relevance by detecting *S. aureus*, a defined non-aureus *Staphylococcus* spp./CoNS target set, high-level mupirocin resistance markers such as *mupA/mupB*, and an internal control. The second well establishes methicillin resistance attribution and *S. aureus* redundancy by detecting *SCCmec*-*orfX* or an equivalent *SCCmec* flanking-region target, *mecA* and, where analytically validated, *mecC*, *spa* or another *S. aureus* confirmatory target, and an internal control.

The reportable category should be generated only after the two wells are interpreted together. *mecA/mecC* without *S. aureus* context should not be reported as MRSA. *SCCmec*-orfX positivity without *mecA/mecC* should be interpreted as an empty cassette or dropout pattern rather than confirmed MRSA. *mupA/mupB* modifies the decolonization pathway but should not, by itself, alter β-lactam prophylaxis. Control failure should produce an invalid result requiring repeat testing or recollection.

Selected *ileS* mutations may be added when the intended use includes low-level mupirocin resistance prediction. *qacA/B* or *qacC* markers may be scientifically informative, but they should remain secondary unless their relationship to chlorhexidine or antiseptic failure is clinically validated. The reason is mechanistic and practical: genotype-to-failure relationships are more direct for *mupA/mupB*-mediated high-level mupirocin resistance than for antiseptic tolerance markers.

The central design principle is organism context assignment. The assay should help determine whether *mecA/mecC* is most consistent with MRSA, methicillin-resistant CoNS, or a mixed staphylococcal population. It should also identify whether an *SCCmec*-*orfX* signal occurs without *mecA/mecC*, consistent with an empty cassette or dropout pattern. These distinctions matter because each molecular state implies a different perioperative action: standard *S. aureus* decolonization, MRSA-directed prophylaxis, alternative decolonization when high-level mupirocin resistance markers are detected, or cautionary interpretation when the molecular pattern is discordant.

### 4.4. Validation Status and Governance of Decision Thresholds

**Figure 1:**
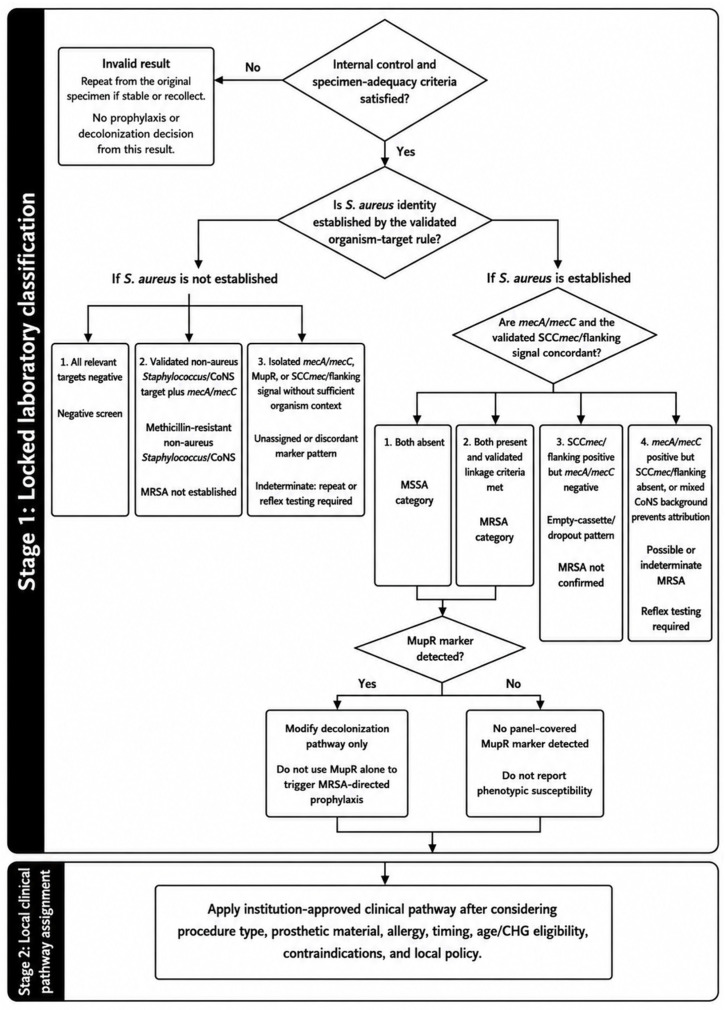
Provisional two-stage algorithm for molecular classification and local clinical pathway assignment. Note. Stage 1 is a locked laboratory classification based on internal control validity and prespecified target combination rules. Stage 2 maps the resulting laboratory category to an institution-approved clinical pathway. Assay-specific positivity thresholds and equivocal or repeat zones must be established during validation. Discordant, mixed, unassigned, or near-cutoff patterns default to repeat or reflex testing rather than automatic pathway assignment. The figure is a development-stage implementation model and not a validated treatment algorithm.

The term “cutoff” encompasses three distinct decisions that should not be conflated. Analytical cutoffs determine whether each individual molecular target is detected, not detected, equivocal, or invalid [54]. These thresholds are platform-specific and must be established from assay validation data [55,56]. Interpretive rules then combine the individual target calls to assign a reportable molecular category [55,56]. Clinical action thresholds finally route a validated molecular category to an institution-approved perioperative pathway [6,7,9]. The present review proposes an interpretive rule structure, but it does not establish universal analytical cutoffs or a validated clinical action threshold. This distinction between analytical calling, reportable molecular classification, and downstream clinical pathway assignment is illustrated in Figure 1.

Validated assay-level calling algorithms exist for specific commercial molecular platforms [55,56]. Such systems use device-specific combinations of *S. aureus*-associated targets, *mecA/mecC*, *SCCmec*-*orfX* or related junction targets, internal controls, and target-specific cycle threshold limits to generate qualitative results [55,56]. However, validation of one commercial platform cannot be transferred to a different multi-target panel. In addition, assay-level validation does not establish the expanded methicillin-resistant CoNS, empty cassette, mixed-population, unassigned resistance marker, or MupR-linked action categories proposed here. Similarly, outcome studies of screen–decolonize–prophylaxis bundles support conventional MRSA/MSSA carrier pathways but do not validate the full molecular taxonomy developed in this review [6,7,9].

Accordingly, the present framework is a provisional categorical rule set rather than a weighted clinical score. Each molecular target must first be called using locked, assay-specific criteria [55,56]. Concordant patterns may then be classified as resolved by the predefined target rule. Patterns with incomplete organism–resistance linkage, mixed-organism signals, target dropout, isolated late amplification, or other discordance must be classified as provisional or indeterminate and subjected to repeat or reflex testing [55,56]. Internal control failure must result in an invalid classification. Raw Ct values should not be interpreted directly by clinicians or converted independently into treatment decisions.

The laboratory information system should generate the reportable category from a version-controlled target combination table. Manual overrides should require documented review by the laboratory medical director and should remain auditable. Even when a molecular category is resolved, pathway assignment remains a separate clinical step. Procedure type, prosthetic material, β-lactam allergy, timing to incision, age or chlorhexidine gluconate eligibility, contraindications, and local policy may modify the final clinical action [6,7,9]. The proposed framework should therefore not activate autonomous medication orders until analytical, clinical, usability, and outcome validation has been completed. The relationship between Stage 1 laboratory classification and Stage 2 local clinical pathway assignment is summarized in Figure 1.

Figure 1 summarizes the sequential decision structure, whereas Table 1 condenses the principal molecular classification states and the disposition required for each state. Detailed target combinations are retained in Appendix A to avoid duplicating the complete molecular truth table in the main text.

### 4.5. Reporting Interface: Converting Genotype into Clinical Action

The reporting interface is where molecular detection becomes clinical action. In preoperative MRSA/MSSA-MupR screening, the assay does not achieve its clinical value simply by identifying targets. Its value emerges when target patterns are mapped onto perioperative decisions that can be executed before incision. The report must therefore operate as an interpretive interface: it should assign resistance signals to the correct organism, separate prophylaxis decisions from decolonization decisions, and make uncertainty visible when the molecular pattern is discordant.

This distinction is essential. A *mecA*-positive signal assigned to *S. aureus* supports an MRSA colonization pathway. A *mecA*-positive signal detected only in coagulase-negative Staphylococcus does not establish MRSA colonization and should not, by itself, trigger MRSA-directed prophylaxis. Similarly, mupirocin resistance markers should redirect the decolonization pathway, but they do not automatically change perioperative β-lactam prophylaxis [6,42,46]. The same molecular signal can therefore imply different actions depending on the organismal background in which it appears.

Accordingly, the report should be organized around clinical action before molecular detail. As shown in Table 2, the report should function as a modular decision interface rather than as a list of detected targets. The top of the report should provide the plain-language molecular classification and classification status, followed by organism–resistance attribution, candidate prophylaxis and decolonization implications, molecular evidence, and repeat or reflex recommendations. This structure allows for each stakeholder to access the layer of information needed for action. Surgeons and anesthesiology teams need a clear preoperative pathway. Pharmacists need separate prophylaxis and decolonization implications. Infection prevention teams need colonization, transmission, adherence, and implementation relevance. Laboratory directors need target-level transparency, organism context assignment, classification status logic, and quality control visibility. Contemporary molecular diagnostic stewardship guidance reinforces this requirement for multidisciplinary governance. Molecular microbiology tests should be evaluated across the preanalytical, analytical, and postanalytical phases, with structured attention to test selection, result interpretation, institutional resources, stakeholder involvement, clinical utility, and monitoring for intended and unintended consequences [54].

Table 2 therefore functions as the report design framework. It specifies what each reporting component is supposed to do, what content should be included, and which clinical user depends on that information. This prevents the report from collapsing into a list of detected targets without clinical hierarchy. The interpretive layer tells the clinical team what the result means. The molecular layer preserves the evidence that justifies that interpretation.

The interpretive logic would need to be standardized and prospectively validated so that each resolved molecular category can be routed reproducibly to an institution-approved clinical pathway, while unresolved patterns default to repeat or reflex testing, as shown in Figure 1. Appendix A provides the expanded target pattern crosswalk for the major combinations of *S. aureus*, non-aureus *Staphylococcus* spp./CoNS, spa, *SCCmec*-*orfX* or *SCCmec*-flanking-region signal, *mecA/mecC*, mupirocin resistance markers, and internal control status. The interpretive logic would need to be standardized and prospectively validated so that each resolved molecular category can be routed reproducibly to an institution-approved clinical pathway, while unresolved patterns default to repeat or reflex testing, as shown in Figure 1. Table 1 summarizes the principal molecular classification states, and Table 2 defines the minimum reporting architecture. The expanded target pattern and candidate pathway crosswalk is retained in Appendix A because these mappings remain hypotheses for prospective validation rather than established clinical action rules. 

The proposed reporting architecture is intended to preserve organism–resistance attribution while making uncertainty explicit. Misclassification remains a central risk: detection of *mecA/mecC* without sufficient organism context may lead to inappropriate MRSA classification, while *SCCmec* flanking positivity without an internal methicillin resistance determinant may result in overcalling MRSA in an empty cassette or dropout pattern [41,42,43]. Similarly, MupR markers should be interpreted only within validated organism context and should modify a decolonization pathway only after appropriate analytical and clinical validation [46,48,49,51]. Accordingly, the framework should be evaluated prospectively for classification accuracy, reflex-testing performance, stewardship effects, usability, and workflow integration before it is used to support institution-approved clinical pathways. 

### 4.6. Cross-Setting Precedents for Molecular Triage

Molecular triage has been implemented in other infectious disease settings when rapid organism or resistance marker detection has been coupled to a prespecified clinical response. Recent multidisciplinary guidance on multiplex infectious disease panels likewise emphasizes that analytical breadth does not itself establish clinical utility; implementation must account for specimen type, patient population, panel coverage, interpretation, workflow fit, and the risk of detecting organisms that may not represent clinically actionable infection [57]. One established example is rapid multiplex molecular identification from positive blood cultures. These systems were developed to identify selected bacterial and fungal organisms and resistance determinants directly from positive blood culture bottles, analytically validated against conventional identification methods, and clinically evaluated within workflows that combined structured interpretive comments with antimicrobial stewardship review. In a randomized trial involving 617 patients, rapid multiplex PCR reduced the median time from Gram stain to organism identification from 22.3 h to 1.3 h, reduced treatment of likely contaminants and broad-spectrum antimicrobial exposure, and produced the fastest antimicrobial escalation or de-escalation when results were coupled to real-time stewardship intervention. Mortality, length of stay, and cost were not significantly different, demonstrating that analytical speed alone does not guarantee improvement in all clinical outcomes [58]. More recent systematic evidence supports the analytical performance of these platforms. A 2025 systematic review and meta-analysis of 74 studies involving 24,590 positive blood culture samples found generally high diagnostic accuracy for organism and antimicrobial resistance detection, while also identifying target-specific limitations and the continuing importance of phenotypic confirmation [59].

A second precedent is molecular point-of-care testing for respiratory viruses. These systems were integrated into emergency department and acute medical unit workflows so that rapid viral identification could inform antiviral treatment, antibiotic reassessment, isolation, and patient placement. In a pragmatic randomized trial of 720 adults, molecular point-of-care testing did not reduce the overall proportion receiving antibiotics or the total duration of antibiotic therapy. It did, however, increase the use of single-dose or brief antibiotic courses, improve appropriate antiviral treatment for influenza, and reduce mean hospital length of stay. A major implementation limitation was that many patients had already received antibiotics before the molecular result became available [60].

These precedents provide several lessons for preoperative MRSA/MSSA-MupR triage. Successful implementation requires more than a rapid analytical result. It requires a locked classification algorithm, structured interpretive language, defined escalation and de-escalation pathways, timely delivery before the relevant clinical decision, stewardship ownership, and prospective measurement of treatment and workflow outcomes. Common pitfalls include detecting a resistance determinant without complete phenotypic susceptibility information, identifying nucleic acid that may not establish clinical causality, limiting interpretation to organisms included on the panel, and delivering results after the treatment decision has already been made. The proposed preoperative framework should therefore be evaluated as a combined diagnostic-and-workflow intervention rather than as an assay alone.

### 4.7. Validation Requirements for the Assay and Integrated Decision System

At present, the proposed framework has not undergone prospective validation as an integrated clinical decision system. Validation of individual molecular targets, or validation of an existing commercial MRSA assay, cannot be assumed to validate the complete multi-target classification and clinical pathway crosswalk presented here. In particular, the classification states and action pathways in Table 1 and the hypothesis-generating target pattern implications in Appendix A have not been calibrated against decolonization failure, antimicrobial use outcomes, surgical site infection, or episode-level outcomes.

Before clinical deployment, the proposed MRSA/MSSA-MupR framework should be validated not only as a molecular assay, but as a clinical decision system [61]. The relevant question is not simply whether the assay detects its targets. The relevant question is whether the assay can support a reliable chain of inference: from organism identity, to methicillin resistance attribution, to mupirocin resistance interpretation, to body site context, to perioperative action. Validation should therefore proceed across linked levels, because failure at any level can break the clinical pathway.

The first level is analytical validation. The assay must establish target-level performance under conditions that approximate the biological complexity of nasal and multisite colonization. This requires limit of detection by target, inclusivity across clinically relevant *S. aureus* lineages and *SCCmec* types, exclusivity against CoNS and non-staphylococcal flora, interference testing, reproducibility, mixed-population challenge panels (i.e., competitive inhibition studies), and evaluation of high-background nasal specimens. The challenge panel should deliberately include methicillin-resistant CoNS, MSSA plus *mecA*-positive CoNS mixtures, empty cassette *S. aureus*, *mecC*-positive strains, *mupA*-positive strains, *mupB*-positive strains when available, and low-level *ileS* resistance variants if such calls are included in the intended use. The target pattern categories in Appendix A should also be stress-tested as part of analytical and interpretive validation, including negative screen, MSSA, MSSA-MupR, MRSA, MRSA-MupR, MR-CoNS without *S. aureus*, MSSA plus MR-CoNS, empty cassette/dropout patterns, *spa* dropout, unassigned *mecA/mecC*, unassigned MupR, mixed staphylococcal populations, internal control failure, and near-cutoff discordance. Each target should also have a prespecified positivity threshold, internal control acceptance criterion, equivocal or repeat interval, invalid result rule, and documented procedure for isolated late amplification; those criteria must be locked before clinical validation begins.

The second level is clinical validation. Molecular interpretation should be compared with clinically relevant comparator methods, including culture, cefoxitin or oxacillin phenotype, PBP2a or equivalent methicillin resistance confirmation, and mupirocin minimum inhibitory concentration. This matters because genotype and phenotype do not always align. Truncated, silent, or nonexpressed resistance determinants can generate discordance between a detected marker and functional resistance [62,63]. The report should therefore distinguish “marker detected” from “phenotypic resistance confirmed” unless phenotype has been directly measured. This distinction is especially important for mupirocin resistance interpretation, where the downstream action may involve withholding mupirocin, selecting an alternative decolonization agent, or triggering pharmacy and infection prevention review.

### 4.8. Decision Rule and Usability Validation

Decision rule validation should be assessed separately from target-level analytical accuracy. The target combination algorithm should be locked before prospective testing. For each reportable category, studies should report agreement with reference microbiology, category-specific positive and negative agreement, misclassification rates, invalid and equivocal frequencies, reflex testing yield, and reproducibility across sites, reagent lots, instruments, specimen operators, and patient populations. Mixed challenge specimens, including empty cassette *S. aureus* combined with *mecA*-positive CoNS, should receive particular attention because coincident target detection does not necessarily establish physical linkage of all signals to the same organism.

Consistency of clinical pathway assignment should be evaluated using standardized molecular result scenarios presented to surgeons, anesthesiologists, pharmacists, infection prevention personnel, and laboratory physicians. Prespecified outcomes should include agreement with the intended pathway, inter-user agreement, time to decision, need for additional explanation, manual override frequency, reflex-testing frequency, and clinically consequential classification or management errors. Any subsequent modification of target cutoffs, target combination rules, report comments, or software logic should be version controlled and should trigger an assessment of whether revalidation is required.

The third level is implementation and outcome validation. A report has clinical utility only if it changes behavior at the preoperative interface. Relevant outcomes include test completion, result turnaround time, vancomycin use, cefazolin preservation, mupirocin avoidance when resistance markers are detected, completion of decolonization, adherence to the prescribed protocol, carriage site distribution when multisite sampling is performed, follow-up clearance, SSI, readmission, length of stay (LOS), and total episode cost. Outcomes should be stratified by MRSA carriage, MSSA carriage, non-carriage, MupR status, and adherence, because orthopedic and spine implementation data suggest that MRSA carriage may remain a residual SSI risk signal even under targeted decolonization, whereas MSSA-associated risk may be mitigated when decolonization is successfully completed [25].

Urgent and trauma-related surgical pathways require separate validation because they change the temporal structure of the problem. In operative fracture fixation, the interval between injury and surgery may be too short for conventional five-day mupirocin decolonization. Incomplete protocol delivery can weaken the practical value of screening even when MRSA/MSSA status is known. Validation studies in these populations should stratify outcomes by time from injury to surgery, open versus closed fracture, staged external-to-internal fixation, MRSA versus MSSA carriage, decolonization completion, number of mupirocin doses received, CHG exposure, prophylaxis selection, SSI organism, and wound healing complications. This is necessary because MRSA carriage may remain a strong SSI risk marker in fracture fixation populations, while the feasibility of full decolonization may be constrained by surgical urgency [26].

When non-mupirocin alternatives are used because of MupR detection, validation should not assume equivalence to mupirocin-based decolonization. Alternative pathways should be evaluated by agent, adherence, recolonization, *S. aureus*/MRSA clinical culture outcomes, SSI outcomes when applicable, adverse events, and local feasibility. This is necessary because alternative nasal agents may not reproduce the anti-staphylococcal effect of mupirocin-based decolonization. Comparative ICU evidence has shown iodophor–CHG to be inferior to mupirocin–CHG for *S. aureus* and MRSA clinical culture outcomes, although that evidence should not be generalized directly to preoperative SSI prevention [8]. The implication is not that alternatives should be avoided. The implication is that alternatives should be locally validated before they are treated as functionally interchangeable [37].

Furthermore, pediatric and neonatal use requires separate implementation validation rather than direct extrapolation from adult surgical pathways. In these distinct settings, validation studies must stratify outcomes by gestational age, chronological age, CHG eligibility, mupirocin completion, discharge before protocol completion, household exposure, spontaneous clearance, MRSA versus MSSA carriage, and infection outcome. Such stratification is necessary because neonatal and young child evidence demonstrates that decolonization effectiveness is frequently constrained by age-specific safety rules, protocol completion, household transmission, and spontaneous clearance, rather than by resistance markers alone [34,36].

For NICU implementation specifically, validation should examine whether the reporting system activates the intended operational pathway. Relevant workflow endpoints include surveillance cadence, reflex culture or PCR workflow, contact precautions, EMR order set activation, staff education, CHG eligibility screening, mupirocin completion, leadership review, and adherence monitoring. Recent NICU implementation studies suggest that MRSA outcomes may improve when surveillance and decolonization protocols are reinforced through system-level compliance mechanisms, whereas parallel MSSA programs may not produce the same outcome effect. This supports an organism-specific and workflow-specific validation strategy rather than assuming that a single MRSA/MSSA report will perform equivalently across populations, organisms, and care settings [35,36].

Also, implementation validation must account for non-molecular causes of decolonization failure. While failure may stem from resistance biology, it is equally likely to reflect multisite, throat, or perineal carriage, as well as household or environmental reservoirs, incomplete adherence, or a poor fit between the laboratory report and the clinical workflow [22]. Therefore, the proposed framework should be evaluated not simply as an analytic test, but as a decision architecture designed to change clinical behavior. In this sense, the report functions as a core component of the intervention by directly shaping how clinicians utilize molecular data.

Because the strongest evidence for body site interaction and resistance-mediated decolonization failure comes from post-discharge MRSA decolonization modeling rather than preoperative surgical screening, these variables should be prospectively evaluated as candidate modifiers of decolonization completion, clearance, recolonization, decolonization decisions, prophylaxis alignment, SSI, and episode-level outcomes. Mechanistic validation should examine whether the report captures body site and strain level variables that may affect decolonization success in surgical pathways. In post-discharge MRSA decolonization data, modeling evidence suggests that nares carriage may function as a central hub for MRSA colonization dynamics, that nasal mupirocin may be a major driver of clearance, and that the full decolonization protocol may operate through interactive effects across multiple body sites [52]. In parallel, resistance modeling suggests that high-level mupirocin resistance may be more strongly associated with decolonization failure than putative chlorhexidine resistance markers or resistance to unrelated antibiotics [53]. Validation studies should therefore test not only whether the assay detects targets accurately, but whether target interpretation supports clinically useful action: identifying site-specific clearance risk, reducing recolonization where feasible, and appropriately redirecting decolonization when high-level MupR markers are present.

Finally, economic validation should avoid simplistic test cost comparisons. A threshold model is more appropriate. The relevant question is not whether the screening test is directly reimbursed. The relevant question is at what colonization prevalence, MRSA SSI rate, MupR prevalence, decolonization failure rate, prophylaxis change rate, adherence rate, screening cost, alternative agent cost, and episode penalty exposure the screening strategy becomes cost-effective or cost-saving [18,22,32]. This approach allows for hospitals to model targeted screening for high-risk procedures before considering universal adoption, while preserving flexibility for pediatric, neonatal, urgent surgery, and institution-specific decolonization pathways.

The validation agenda follows directly from the central thesis of this review. A next-generation MRSA/MSSA-MupR assay should not be judged only by whether it detects genes. It should be judged by whether it improves inference and action: assigning resistance to the correct organism, separating prophylaxis from decolonization decisions, identifying decolonization failure risk, fitting the surgical timeline, and producing measurable stewardship, infection prevention, and episode-level value.

## 5. Limitations

### Limitations and Research Agenda

Several limitations define the boundary conditions of this review. First, colonization is not infection. A positive nasal or multisite screen identifies a risk state, but it does not prove that a later SSI would be caused by the same organism, nor that screening alone would prevent infection. MRSA/MSSA-MupR screening should therefore be understood as one component of a prevention system, not as an independent SSI prevention solution.

Second, genotype is not always phenotype. *mupA* is strongly associated with high-level mupirocin resistance, and *mupB* is a recognized alternative mechanism, but low-level *ileS* interpretation requires defined variant coverage, phenotype correlation, and careful reporting. The report should distinguish “resistance marker detected” from “phenotypic resistance confirmed” unless mupirocin MIC or another validated phenotypic method has been performed [62]. A further central limitation is that no externally validated composite score, universal molecular cutoff, or prospectively validated target-to-action decision rule currently supports the full framework. The original qualitative confidence descriptors should not be interpreted as calibrated probabilities of colonization, decolonization failure, or surgical site infection. Analytical positivity thresholds and equivocal zones will also vary by platform, instrument, specimen preparation, and target chemistry. Accordingly, the framework cannot currently support autonomous clinical pathway assignment and requires assay-specific analytical validation, prospective category-level clinical validation, and local implementation testing.

Third, local context determines value. The utility of distinguishing MRSA, MSSA, CoNS-associated *mecA*, empty cassette patterns, and MupR depends on local prevalence, procedure mix, baseline SSI risk, prophylaxis policy, and decolonization performance. A hospital with low MupR prevalence and reliable decolonization outcomes may not need the same reporting architecture as a hospital with prevalent MupR MRSA clones.

Fourth, decolonization failure is not exclusively driven by molecular factors. Even in the absence of high-level mupirocin resistance markers, failure can result from multisite, throat, or perineal carriage, as well as household transmission, environmental contamination, poor adherence, skin integrity issues, or overly burdensome protocols [22,64]. While a molecular report can successfully optimize agent selection, it ultimately cannot guarantee the operational execution of the decolonization pathway.

Fifth, non-mupirocin alternatives are not automatically equivalent. ICU evidence comparing iodophor–CHG with mupirocin–CHG should not be generalized directly to preoperative SSI prevention, but it cautions against assuming that povidone-iodine, alcohol-based nasal antisepsis, photodisinfection, or other alternatives can simply replace mupirocin without local validation [8,38]. When alternatives are used because of MupR detection, institutions should monitor adherence, recolonization, adverse events, *S. aureus*/MRSA clinical cultures, SSI outcomes when applicable, and feasibility [65].

Sixth, population boundaries must be carefully considered. Evidence derived from neonatal and pediatric populations should not be directly generalized to adult surgical pathways. Factors such as prematurity, CHG eligibility, discharge timing, spontaneous clearance, household transmission, outbreak control goals, and age-specific safety constraints fundamentally alter the clinical implications of a positive test result [34]. Furthermore, NICU data indicate that MRSA and MSSA programs may not yield identical effects, underscoring the need for organism-specific workflow evaluations [34,35].

Seventh, a portion of the supporting evidence remains preliminary or indirect. For instance, orthopedic and spine data connecting MRSA/MSSA carriage, targeted decolonization, restricted vancomycin use, and SSI outcomes should be viewed as hypothesis-generating when limited to conference abstract evidence [25]. Furthermore, computational models that support body site-aware and mupirocin resistance-aware reporting are currently based on post-discharge MRSA decolonization data rather than direct preoperative surgical screening trials [52,53]. Finally, although recent findings identify MRSA carriage as a strong SSI risk marker in fracture fixation, the operational reality of urgent trauma pathways frequently precludes the completion of standard five-day mupirocin protocols [26].

Finally, while this triage framework is optimized for elective adult surgical episodes, it is not inherently exhaustive. Decolonization protocols must be tailored not only to the organism but to the host’s physiology, with neonatal and pediatric settings requiring evidence that is distinct from adult surgical standards [66]. Future iterations of this model should explore the integration of age-specific microbial data to ensure that triage logic remains clinically safe across the entire patient spectrum.

These limitations do not weaken the framework; they define the validation agenda. Future studies should test whether interpretive MRSA/MSSA-MupR reporting improves three linked outcomes: antimicrobial stewardship, decolonization stewardship, and value-based episode performance [67]. The most useful studies would compare standard MRSA/MSSA reporting with interpretive molecular triage and measure prophylaxis selection, vancomycin use, cefazolin preservation, decolonization changes, mupirocin avoidance when resistance markers are detected, adherence, SSI, readmission, LOS, pharmacy cost, and total episode cost.

A second research stream should test usability. Surgeons, anesthesiologists, pharmacists, infection preventionists, laboratory professionals, and administrators should be evaluated using standardized result scenarios to determine whether they select the intended local pathway consistently without additional explanation. Such studies should report inter-user agreement, κ statistics, time to decision, manual override rates, requests for clarification, and clinically consequential errors. A third stream should test population-specific implementation in orthopedic, cardiothoracic, trauma, ICU, neonatal, and pediatric settings. The same molecular result may require different action depending on age, procedure type, local epidemiology, surgical timing, and institutional decolonization policy.

## 6. Conclusions

Preoperative MRSA/MSSA screening may benefit from greater interpretive resolution than binary positive or negative reporting, particularly when organism identity, methicillin resistance attribution, mixed staphylococcal populations, and mupirocin resistance markers may have different clinical implications. This review proposes a development-stage molecular classification and reporting architecture for organizing these findings. The proposed framework has not been prospectively validated as an integrated clinical decision system and should not be interpreted as an established treatment algorithm.

The potential benefits of molecular triage, including improved alignment of perioperative prophylaxis, reduced unnecessary antimicrobial exposure, more appropriate decolonization selection, improved workflow coordination, and lower episode-level cost, remain hypothetical. These outcomes must be evaluated in prospective comparative studies measuring surgical site infection, antimicrobial utilization, decolonization completion and clearance, adverse events, readmission, length of stay, workflow performance, and healthcare costs.

Future studies should establish assay-specific analytical thresholds, validate target combination and organism attribution rules, assess reproducibility and clinical usability, and determine whether integration with institution-approved perioperative pathways improves patient and stewardship outcomes. Until such evidence is available, the proposed framework should be regarded as a hypothesis-generating reporting and validation architecture rather than a validated scoring system, universal molecular cutoff scheme, or stand-alone clinical strategy. 

## Figures and Tables

**Table 1:** Provisional molecular classification states and required disposition.

Molecular Classification State	Representative Molecular Pattern	Classification Status	Required Disposition
Negative screen	All reportable organism and resistance-marker targets negative; internal control valid	Resolved by predefined rule	No MRSA-specific action based on this assay. Follow routine institutional perioperative policy.
MSSA	*S. aureus* identity established; *mecA/mecC* and *SCCmec*/flanking targets absent	Resolved after assay-specific validation	Apply the institution-approved MSSA or standard perioperative pathway. Evaluate MupR separately for decolonization.
MRSA	*S. aureus* identity established; *mecA/mecC* and *SCCmec*/flanking targets concordant under a validated linkage rule	Resolved after assay-specific validation	Apply the institution-approved MRSA colonization pathway. Evaluate MupR separately for decolonization.
Methicillin-resistant non-aureus *Staphylococcus*/CoNS	Validated non-aureus staphylococcal target and *mecA/mecC* detected without established *S. aureus* identity	Resolved only if organism attribution is validated	Do not classify as MRSA or automatically activate MRSA-directed prophylaxis.
Provisional or indeterminate pattern	Empty cassette/dropout pattern, mixed staphylococcal population, discordant *S. aureus* targets, isolated resistance marker, or insufficient organism–resistance linkage	Provisional or indeterminate	Repeat or perform reflex testing when the result would change perioperative management. Do not assign an automatic clinical pathway.
Invalid result	Internal control failure, inhibition, required-well failure, or unresolved analytical failure	Invalid	Repeat from the original specimen if stable or recollect. Do not use the result for prophylaxis or decolonization decisions.

Note. Classification status is not a probability, clinical risk score, or estimate of surgical site infection risk. “Resolved” means that a prespecified target combination rule has been satisfied after assay-specific validation. “Provisional” or “indeterminate” means that the molecular pattern does not provide sufficient organism–resistance linkage for autonomous clinical pathway assignment. Numeric Ct, fluorescence, and signal ratio thresholds are assay- and instrument-specific and are not established by this review. MupR status modifies the decolonization pathway and should not independently trigger MRSA-directed prophylaxis.

**Table 2:** Minimum reporting architecture for preoperative MRSA/MSSA-MupR molecular screening.

Reporting Component	Required Content	Primary Purpose
Reportable classification	Negative, MSSA, MRSA, methicillin-resistant non-aureus *Staphylococcus*/CoNS, provisional or indeterminate, or invalid	Provides the primary laboratory interpretation
Classification status	Resolved, provisional, indeterminate, or invalid	Makes uncertainty explicit and prevents overcalling
Prophylaxis implication	Routine pathway, institutional MRSA pathway, or no automatic pathway change	Separates organism classification from perioperative antibiotic action
Decolonization implication	MupR marker detected or not detected, with local pathway language	Clarifies whether mupirocin-based decolonization remains reasonable
Reflex recommendation	Repeat, culture, phenotypic confirmation, or orthogonal molecular testing when indicated	Directs management of discordant or unresolved patterns
Algorithm governance	Assay version, rule version, internal control status, override documentation, and effective date	Ensures consistent, auditable classification

Note. The report should present the interpreted category before raw target data. Molecular findings should be converted into a reportable classification by a locked, version-controlled algorithm. Clinical actions remain subject to institution-approved prophylaxis and decolonization policies. Raw Ct values should not be used independently to assign treatment.

## Data Availability

No new data were created or analyzed in this study. Data sharing is not applicable to this article.

## Abbreviations

The following abbreviations are used in this manuscript:

AMR	Antimicrobial resistance
CHG	Chlorhexidine gluconate
CoNS	Coagulase-negative staphylococci
DMID	Diagnostic Microbiology and Infectious Disease
EMR	Electronic medical record
HAC	Hospital-acquired condition
HACRP	Hospital-Acquired Condition Reduction Program
HRRP	Hospital Readmissions Reduction Program
*ileS*	Native staphylococcal isoleucyl-tRNA synthetase gene (low-level mupirocin resistance)
LOS	Length of stay
*mecA*	Gene encoding an alternative penicillin-binding protein associated with methicillin resistance
*mecC*	*mecA* homolog associated with methicillin resistance
MRSA	Methicillin-resistant *Staphylococcus aureus*
MSSA	Methicillin-susceptible *Staphylococcus aureus*
*mupA*	Plasmid-associated gene encoding high-level mupirocin resistance
*mupB*	Gene encoding an alternative high-level mupirocin-resistance mechanism
MupHR	High-level mupirocin resistance
MupLR	Low-level mupirocin resistance
MupR	Mupirocin resistance or mupirocin-resistance marker detected
NAAT	Nucleic acid amplification testing
*orfX*	*Staphylococcus aureus* chromosomal open reading frame used as the *SCCmec* integration junction target
PharmD	Doctor of Pharmacy/pharmacist
pre-op	Preoperative
*S. aureus*	*Staphylococcus aureus*
*SCCmec*	Staphylococcal cassette chromosome *mec*
*SCCmec*-*orfX*	Molecular junction between *SCCmec* and the *S. aureus orfX* integration site
*spa*	Staphylococcal protein A gene
spp.	Species plural
SSI	Surgical site infection, or surgical-site infection
TJA	Total joint arthroplasty
VAP	Ventilator-associated pneumonia
β-lactam	Beta-lactam antimicrobial class

## Appendix A. Detailed Hypothesis-Generating Target Matrix

**Table A1:** Quality control failures and unassigned molecular signals.

ID	Target Profile	Provisional Molecular Classification	Interpretive Rationale	Classification Status	Required Validation Disposition
1	±/±/±/±/±/±/Fail	Invalid result	Internal control failure may reflect inhibition, extraction failure, reagent failure, or inadequate specimen integrity.	Invalid	Do not assign a molecular or clinical pathway. Repeat from the original specimen if stable or recollect.
2	±/±/±/±/±/±/One well fails	Partial invalid or incomplete result	Targets in the failed well cannot be interpreted reliably.	Invalid or incomplete	Do not infer MRSA, MSSA, CoNS, or MupR status from the failed target set. Repeat the affected well or recollect.
3	−/−/−/−/−/−/Pass	Negative screen	No panel-covered organism or resistance marker target is detected.	Resolved only after assay-specific validation	A routine local pathway may be considered only after the negative classification rule has been validated.
4	−/−/−/−/+/−/Pass	Unassigned methicillin resistance marker	*mecA/mecC* is detected without sufficient organism context.	Indeterminate	Do not classify as MRSA or methicillin-resistant CoNS. Repeat or perform reflex testing when clinically consequential.
5	−/−/−/−/−/+/Pass	Unassigned mupirocin resistance marker	A MupR marker is detected without sufficient organism attribution.	Indeterminate	Do not report mupirocin-resistant *S. aureus*. Repeat or perform reflex testing if decolonization selection would change.
6	−/−/−/+/−/±/Pass	Isolated *SCCmec* flanking signal	The *SCCmec*-flanking target is detected without *S. aureus* or *mecA/mecC* support.	Indeterminate	MRSA is not established. Repeat or perform reflex testing for persistent, near-cutoff, or clinically consequential results.
7	−/−/−/+/+/±/Pass	Possible MRSA with organism target dropout	*SCCmec*-flanking and *mecA/mecC* signals are present without validated *S. aureus* identity.	Indeterminate	Report that MRSA cannot be established or excluded. Reflex culture or orthogonal molecular confirmation is required.

**Table A2:** Non-aureus staphylococcal patterns.

ID	Target Profile	Provisional Molecular Classification	Interpretive Rationale	Classification Status	Required Validation Disposition
8	+/−/−/−/−/−/Pass	Non-aureus Staphylococcus detected; MRSA/MSSA not detected	A validated non-aureus staphylococcal target is detected without *S. aureus* or resistance markers.	Resolved only if the non-aureus target set is validated	Do not classify as MRSA or MSSA. Any clinical relevance remains organism- and protocol-specific.
9	+/−/−/−/+/−/Pass	Methicillin-resistant non-aureus Staphylococcus detected	*mecA/mecC* is detected within a non-aureus staphylococcal background.	Resolved only if organism attribution is validated	Do not classify as MRSA. Reflex testing may be considered if the organism or resistance attribution would alter management.
10	+/−/−/−/−/+/Pass	MupR marker in a non-aureus staphylococcal background	A MupR marker is detected without *S. aureus.*	Provisional unless organism-specific MupR attribution is validated	Do not report mupirocin-resistant S. aureus. The result may inform future decolonization studies but should not independently direct treatment.
11	+/−/−/−/+/+/Pass	Methicillin and mupirocin resistance markers in a non-aureus background	*mecA/mecC* and MupR are detected with a non-aureus staphylococcal target.	Provisional	Do not classify as MRSA. Organism-specific attribution and phenotypic correlation are required.
12	+/−/−/+/−/±/Pass	Non-aureus Staphylococcus with isolated *SCCmec* flanking signal	The *SCCmec*-associated signal lacks both *S. aureus* and *mecA/mecC* support.	Indeterminate	Do not establish MRSA. Repeat or reflex when the pattern persists or would change management.
13	+/−/−/+/+/±/Pass	Complex non-aureus staphylococcal pattern; MRSA cannot be excluded	Non-aureus Staphylococcus, *SCCmec* flanking, and *mecA/mecC* are detected without direct *S. aureus* support.	Indeterminate	Reflex culture or orthogonal molecular testing is required before assigning MRSA status.

**Table A3:** *S. aureus*-positive patterns with concordant organism targets.

ID	Target Profile	Provisional Molecular Classification	Interpretive Rationale	Classification Status	Required Validation Disposition
14	−/+/+/−/−/−/Pass	MSSA; MupR marker not detected	Concordant *S. aureus* targets are present without panel-covered methicillin or mupirocin resistance markers.	Resolved only after the complete MSSA rule is validated	May enter an institution-approved MSSA pathway only after analytical and clinical validation.
15	−/+/+/−/−/+/Pass	MSSA with MupR marker	*S. aureus* is detected without methicillin resistance markers, but a MupR marker is present.	Resolved only if MupR attribution to *S. aureus* is validated; otherwise provisional	MupR may modify a validated decolonization pathway but should not independently trigger MRSA-directed prophylaxis.
16	−/+/+/+/−/−/Pass	Empty cassette or dropout pattern; MRSA not confirmed	*S. aureus* and *SCCmec*-flanking targets are present without *mecA/mecC*.	Provisional	Do not classify as MRSA. Reflex testing is required when the distinction would alter management.
17	−/+/+/+/−/+/Pass	Empty cassette or dropout pattern with MupR marker	An MSSA-like methicillin pattern co-occurs with *SCCmec*-flanking and MupR signals.	Provisional	MRSA is not confirmed. MupR attribution and decolonization implications require validation or phenotypic confirmation.
18	−/+/+/+/+/−/Pass	MRSA; MupR marker not detected	Concordant *S. aureus*, *SCCmec*-flanking, and *mecA/mecC* signals support *S. aureus*-linked methicillin resistance.	Resolved only after the complete MRSA linkage rule is validated	May enter an institution-approved MRSA pathway only after assay-specific validation. Absence of MupR does not prove phenotypic mupirocin susceptibility.
19	−/+/+/+/+/+/Pass	MRSA with MupR marker	Concordant MRSA-associated signals are accompanied by a MupR marker.	Resolved only if both MRSA and MupR attribution criteria are validated	MRSA and decolonization implications must remain separate. MupR should modify only a validated decolonization branch.
20	−/+/+/−/+/−/Pass	Possible MRSA with *SCCmec*-flanking target dropout	*S. aureus* and *mecA/mecC* are detected without *SCCmec* flanking support.	Indeterminate	Do not force an MSSA or MRSA classification. Reflex testing is required.
21	−/+/+/−/+/+/Pass	Possible MRSA with *SCCmec*-flanking dropout and MupR marker	*S. aureus*, *mecA/mecC*, and MupR are detected without *SCCmec* flanking support.	Indeterminate	Confirm methicillin-resistance attribution and MupR attribution before clinical pathway assignment.

**Table A4:** *S. aureus* organism-target discordance.

ID	Target Profile	Provisional Molecular Classification	Interpretive Rationale	Classification Status	Required Validation Disposition
22	−/+/−/−/−/−/Pass	*S. aureus* primary target detected with spa dropout	The primary *S. aureus* target is detected without spa or resistance marker support.	Provisional	Resolve according to the validated organism target rule. Repeat or reflex if near cutoff or clinically consequential.
23	−/+/−/−/−/+/Pass	*S. aureus* primary target with spa dropout and MupR marker	*S. aureus* identity is supported by one target, with reduced redundancy and a MupR signal.	Provisional	Confirm *S. aureus* and MupR attribution before assigning a decolonization implication.
24	−/+/−/+/−/±/Pass	*S. aureus* primary target with spa dropout and empty cassette pattern	The primary *S. aureus* and *SCCmec* flanking targets are present without spa or *mecA/mecC*.	Indeterminate	MRSA is not confirmed. Repeat or reflex when management would change.
25	−/+/−/+/+/±/Pass	Probable MRSA with spa dropout	The primary *S. aureus* target, *SCCmec* flanking, and *mecA/mecC* are present, but spa is absent.	Provisional or resolved only under a validated dropout rule	Report as MRSA only if the assay-specific algorithm validates this pattern. Otherwise reflex.
26	−/−/+/−/−/−/Pass	spa-only presumptive *S. aureus* signal	spa is detected without the primary *S. aureus* target or resistance marker support.	Indeterminate	Repeat or reflex unless spa alone has been validated as sufficient for organism classification.
27	−/−/+/−/−/+/Pass	spa-only signal with MupR marker	A presumptive *S. aureus* signal and MupR marker are present without the primary organism target.	Indeterminate	Confirm organism and MupR attribution before reporting a decolonization implication.
28	−/−/+/+/−/±/Pass	spa-only signal with possible empty cassette pattern	spa and *SCCmec* flanking are detected without the primary *S. aureus* target or *mecA/mecC*.	Indeterminate	MRSA is not established. Repeat or perform reflex testing.
29	−/−/+/+/+/±/Pass	Probable MRSA with primary *S. aureus* target dropout	spa, *SCCmec* flanking, and *mecA/mecC* support an MRSA-like pattern, but the primary *S. aureus* target is absent.	Provisional or resolved only under a validated dropout rule	Report as MRSA only if this exact combination has been validated. Otherwise reflex.

**Table A5:** Mixed *S. aureus* and non-aureus staphylococcal patterns.

ID	Target Profile	Provisional Molecular Classification	Interpretive Rationale	Classification Status	Required Validation Disposition
30	+/+/+/−/−/−/Pass	MSSA with non-aureus *Staphylococcus* co-detection	Concordant *S. aureus* targets are present with a non-aureus staphylococcal background and no resistance markers.	Resolved only after both organism rules are validated	The non-aureus signal should not independently alter the validated MSSA classification or pathway.
31	+/+/+/−/−/+/Pass	MSSA and non-aureus *Staphylococcus* with MupR marker	*S. aureus* and non-aureus staphylococci are present, but the source of the MupR marker may be uncertain.	Provisional	Do not assign the MupR marker to *S. aureus* without validated organism-specific linkage.
32	+/+/+/−/+/−/Pass	MSSA with methicillin-resistant non-aureus *Staphylococcus*; MRSA not confirmed	*S. aureus*, non-aureus *Staphylococcus*, and *mecA/mecC* are detected without *SCCmec*-flanking support.	Indeterminate	The pattern may represent MSSA plus methicillin-resistant CoNS or MRSA target dropout. Reflex testing is required.
33	+/+/+/−/+/+/Pass	Mixed population with unassigned methicillin and mupirocin resistance markers	*S. aureus* and non-aureus staphylococci are present with *mecA/mecC* and MupR, but organism linkage is unresolved.	Indeterminate	Do not assign MRSA or MupR status to *S. aureus* without confirmatory linkage.
34	+/+/+/+/−/−/Pass	MSSA empty cassette pattern with non-aureus *Staphylococcus*	*S. aureus* and non-aureus staphylococci are detected with *SCCmec* flanking but without *mecA/mecC*.	Provisional	MRSA is not confirmed. Reflex testing is required when the result would alter management.
35	+/+/+/+/−/+/Pass	Empty cassette mixed population with MupR marker	The pattern contains *S. aureus*, non-aureus *Staphylococcus*, *SCCmec* flanking, and MupR without *mecA/mecC*.	Provisional	Confirm MupR attribution and maintain MRSA-not-confirmed status unless reflex testing supports otherwise.
36	+/+/+/+/+/−/Pass	MRSA with non-aureus *Staphylococcus* co-detection	Concordant MRSA-associated targets are present in a mixed staphylococcal background.	Resolved only under a validated mixed-population linkage rule	May enter a local MRSA pathway only if the algorithm demonstrates adequate *S. aureus*–methicillin resistance linkage.
37	+/+/+/+/+/+/Pass	MRSA and non-aureus *Staphylococcus* with MupR marker	An MRSA-like pattern and MupR marker occur in a mixed staphylococcal specimen.	Resolved only if MRSA and MupR attribution are both validated	Do not assume the MupR marker belongs to MRSA without organism-specific attribution.
38	+/+/−/−/−/±/Pass	Mixed staphylococcal population with spa dropout; methicillin resistance not detected	The primary *S. aureus* and non-aureus targets are present, but spa is absent.	Provisional	Resolve according to the validated organism-target rule. Confirm MupR attribution if the marker is present.
39	+/+/−/+/+/±/Pass	Probable MRSA in a mixed population with spa dropout	The primary *S. aureus* target, *SCCmec* flanking, and *mecA/mecC* are present with a non-aureus background, but spa is absent.	Provisional or indeterminate	Report as MRSA only if the mixed-population and spa-dropout combination has been validated. Otherwise reflex.
40	+/−/+/−/−/±/Pass	spa-positive *S. aureus* pattern with primary target dropout and non-aureus co-detection	spa and a non-aureus target are present without the primary *S. aureus* target.	Indeterminate	Confirm *S. aureus* identity before assigning an MSSA or decolonization pathway.
41	+/−/+/+/+/±/Pass	Probable MRSA with primary *S. aureus* target dropout in a mixed population	spa, *SCCmec* flanking, *mecA/mecC*, and a non-aureus target are present without the primary *S. aureus* target.	Indeterminate	Reflex confirmation is required because mixed-organism DNA complicates resistance attribution.
42	+/+ or −/+ or −/+/+/+/Pass	Complex mixed staphylococcal population with methicillin- and mupirocin-resistance markers	Multiple organism and resistance signals are detected; *S. aureus* linkage is not necessarily established.	Indeterminate unless all linkage criteria are validated	Assign MRSA and MupR classifications only when the locked algorithm establishes organism-specific linkage. Otherwise report as indeterminate and reflex.

Appendix A is a development-stage Boolean truth table rather than an externally validated scoring system. It should not be used to infer a numerical probability of MRSA colonization, decolonization failure, or surgical site infection. Before clinical use, each positive, negative, equivocal, and invalid target state must be generated using locked, assay-specific calling criteria. Equivocal, isolated late amplification, mixed, or persistently discordant signals must not be forced into a resolved category. Manual overrides should require laboratory medical director review and should be recorded for audit and revalidation.
